# Development of the patient-oriented research curriculum in child health (PORCCH)

**DOI:** 10.1186/s40900-021-00276-z

**Published:** 2021-05-10

**Authors:** Colin Macarthur, Catharine M. Walsh, Francine Buchanan, Aliza Karoly, Linda Pires, Graham McCreath, Nicola L. Jones

**Affiliations:** 1 SickKids Research Institute, Hospital for Sick Children, Toronto, Canada; 2 Department of Paediatrics, Faculty of Medicine, University of Toronto, Toronto, Canada; 3 Division of Gastroenterology, Hepatology and Nutrition, Hospital for Sick Children, Toronto, Canada; 4 SickKids Learning Institute, Hospital for Sick Children, Toronto, Canada; 5 The Wilson Centre, Faculty of Medicine, University of Toronto, Toronto, Canada; 6 Family Advisor, Hospital for Sick Children, Toronto, Canada; 7 Institute of Health Policy, Management, and Evaluation, University of Toronto, Toronto, Canada; 8 Canadian Child Health Clinician Scientist Program, Toronto, Canada; 9 Department of Physiology, University of Toronto, Toronto, Canada

**Keywords:** Patient-oriented research, Child Health Research, Education, Capacity development

## Abstract

**Background:**

The Canadian Institutes for Health Research launched a national ‘Strategy for Patient-Oriented Research’ (SPOR) in 2011. Patient-oriented research is defined as a continuum of research that engages patients as partners, focuses on patient-identified priorities, and improves patient outcomes. Capacity development is a core element of SPOR. Barriers to patient-oriented research include unfamiliarity with the research process for patients and families and unfamiliarity with the methods of patient and family engagement for researchers.

**Methods:**

The aim of the Patient-Oriented Research Curriculum in Child Health (PORCCH) is to build capacity in patient-oriented research in child health among patients and families, researchers, healthcare professionals, decision-makers, and trainees through a curriculum delivered via a series of interactive online modules (e-learning). A multi-disciplinary, multi-stakeholder steering committee, which included patients and families, guided the development of the curriculum and provided feedback on individual modules. The content, design, and development of each module were co-led by a parent and researcher in an equal partnership.

**Results:**

PORCCH consists of a series of five modules. All modules are interactive and include video vignettes and knowledge comprehension questions. Access to the modules is free and each module takes approximately 30 min to complete. The five modules are: Research 101 (an Introduction to Patient-Oriented Research, parts 1 and 2), Patient Engagement 101 (an Introduction to Patient Engagement in Child Health Research, parts 1 and 2), and Research Ethics 101.

**Conclusions:**

PORCCH was developed specifically to overcome recognized barriers to the engagement of patients and families in child health research. The aim of the curriculum is to build capacity in patient-oriented research in child health. The goal is for PORCCH to be a useful resource for all stakeholders involved in patient-oriented research: patients and families, researchers, healthcare professionals, decision-makers, and trainees.

## Plain English summary

Canada launched a national ‘Strategy for Patient-Oriented Research’ in 2011. The aim of the strategy is to improve patients’ health and well being by focusing on research that is important to patients, and by involving patients and families as equal partners in research. Our group developed the Patient-Oriented Research Curriculum in Child Health (PORCCH) to help patients and families, and other individuals involved in research, learn about patient-oriented research. Patients and families will learn about the research process, who is involved, and the timelines of a research study. Researchers will learn about the methods used in patient-oriented research and some practical ways to meaningfully engage patients and families in research. The online curriculum includes five modules. The design and development of each module was co-led by a parent and researcher in an equal partnership. Access to the modules is free (www.porcch.ca) and each module takes approximately 30 min to complete. The five modules are: a) Research 101 part 1, What is Health Research and Who is Involved? b) Research 101 part 2, Timeline of a Research Study, c) Patient Engagement 101 part 1, Foundations of Patient Engagement, d) Patient Engagement 101 part 2, Patient Engagement in Practice, and e) Research Ethics 101. It is hoped that PORCCH will be a useful resource for patients and families and other stakeholders involved in patient-oriented research in child health.

## Background

The Canadian Institutes for Health Research (CIHR) launched a national ‘Strategy for Patient-Oriented Research’ (SPOR) in 2011 [1]. CIHR defines patient-oriented research as a continuum of research that engages patients as partners, focuses on patient-identified priorities, and improves patient outcomes. The word ‘patient’ is used as an overarching term inclusive of individuals with personal experience of a health issue as well as informal caregivers, including family and friends. The vision for SPOR is to improve patient outcomes through the integration of research evidence into practice, policy, and health system improvement.

SPOR comprises five core elements: a) SUPPORT Units (multi-disciplinary research support centres located in provinces and territories across Canada); b) National Research Networks (for example, CHILD-BRIGHT a pan-Canadian research network focused on improving outcomes for children with brain-based developmental disabilities); c) Clinical Trials (creation of a Canadian Coordinating Centre and innovative clinical trials initiatives); d) Patient Engagement (enabling patients as equal partners in the research process); and e) Capacity Development.

All SPOR elements have a responsibility for capacity development in patient-oriented research. Guiding principles articulated in the SPOR Capacity Development Framework include ensuring that patients have the capability and support to meaningfully contribute to and participate in research, that all participants in patient-oriented research receive the proper training and support, and that access to such training and support should be equitable [2].

Patient-oriented research, given its focus on patient-identified priorities, perspectives, and outcomes, is hypothesized to improve the relevance, quality, and uptake of health research [3]. There is empirical evidence, albeit limited, of a positive impact of patient-oriented research on the research process as well as on the experience of the research team and patient partners [4–7]. The published literature, however, has identified that a lack of training, skills, and knowledge in patient-oriented research – among both researchers and patients and families – are barriers to the effective practice of patient-oriented research [8–11]. The purpose of this paper is to describe the development of a curriculum to support capacity development in patient-oriented research in child health.

## Methods

### Development of the patient-oriented research curriculum in child health (PORCCH)

Funding for the development of the Patient-Oriented Research Curriculum in Child Health (PORCCH) was secured through a peer-reviewed SPOR funding competition for capacity development in patient-oriented research. Key partners provided additional funds and input (see Acknowledgments). The cost to produce each module was approximately $20,000 CAD.

The aim of PORCCH is to build capacity in patient-oriented research in child health among patients and families, researchers, healthcare professionals, decision-makers, and trainees through a curriculum delivered via a series of interactive online modules (e-learning). A multi-disciplinary, multi-stakeholder steering committee was struck to guide the development of the curriculum and provide regular input and feedback on the individual modules as they were developed. The steering committee included representation from patients and families, family advisory networks, SPOR SUPPORT Units, SPOR Research Networks, clinical research, clinical pediatrics, pedagogy, knowledge translation, and instructional design. The initial aim was for the steering committee to meet quarterly, at minimum, to provide feedback on module development. The parents on the steering committee and the parents involved in co-leading the development of the modules – all of whom had lived experience with a child with a long-term condition - were selected from established Family Advisory Networks at two children’s hospitals.

A series of five PORCCH modules were approved by the steering committee. The content, design, and development of each module were co-led by a parent and a researcher working together in an equal partnership. A key guiding principle for module development was ensuring that the modules were evidence-informed. Module co-leads were responsible for identifying and reviewing the relevant literature (with support from the steering committee) and incorporating the pertinent evidence into the module.

Module co-leads met regularly over several months to draft module content and layout. Throughout the development process, the co-leads received feedback on module content, style, and design from the steering committee at quarterly meetings. Patients and families provided specific input on issues such as accessibility, inclusiveness, pragmatic aspects of research partnership, and how to achieve “authentic and meaningful” research partnership. Additionally, formal usability testing was carried out with end users to refine module content and design to optimize learner experience. This iterative development process took up to a year to finalize each module. Final production of the modules was out-sourced to a third party vendor.

The intent of PORCCH is to build capacity by increasing the “patient-oriented research readiness” of all stakeholders. In that context, peer-reviewed funding from CIHR has also been secured to evaluate PORCCH (SPOR Patient-Oriented Research Collaboration Grant #397481). A series of studies, using a mixed methods approach, are underway to evaluate the usability of PORCCH and the impact of the curriculum on stakeholder self-efficacy and knowledge.

## Results

### PORCCH modules

All PORCCH modules are interactive and include video vignettes and knowledge comprehension questions. Access to the modules is free and a certificate is provided on completion of each module. Each module takes approximately 30 min to complete. PORCCH modules may be found at www.porcch.ca.

*Research 101* - an Introduction to Patient-Oriented Research – parts 1 and 2 were the first two modules developed. The Research 101 modules are targeted particularly towards patients and families, and individuals without a formal background in research, although the modules may also be of value to decision-makers and other stakeholders with modest experience in research.

Research 101 part 1 is titled: “What is Health Research and Who is Involved?” On completion of Research 101 part 1, users will:

- Be able to define health research and patient-oriented research,
- Understand the value of patient engagement in health research,
- Be familiar with the key players in health research, and
- Understand the difference between a research participant and a research partner.

Research 101 part 2 is titled: “Timeline of a Research Study.” On completion of Research 101 part 2, users will:

- Be familiar with the key steps in a research study,
- Understand how patients and families can engage as partners throughout the timeline of a research study,
- Understand the potential impact of health research, and
- Be familiar with the challenges and benefits of patient-oriented research.

*Patient Engagement 101* – an Introduction to Patient Engagement in Child Health Research – also consists of two modules - parts 1 and 2. The modules provide an overview of the key concepts and practical aspects of effective patient and family engagement in research and are particularly targeted towards researchers and clinicians. The modules, however, are also relevant and applicable to patients and families, clinicians, decision-makers and other stakeholders involved in patient-oriented research.

Patient Engagement 101 part 1 is titled: “Foundations of Patient Engagement.” On completion of Patient Engagement 101 part 1, users will be familiar with:

- International initiatives promoting patient and public involvement in health research,
- Values and goals of patient engagement in health research, and
- Key elements of effective patient engagement in health research.

Patient Engagement 101 part 2 is titled: “Patient Engagement in Practice.” On completion of Patient Engagement 101 part 2, users will be familiar with the research evidence on patient engagement and how it informs:

- Practical aspects of patient engagement,
- Challenges of practicing patient engagement, and
- Various methods of patient engagement.

The final module in the PORCCH series is on Research Ethics 101. This module is a high-level introduction to research ethics that will be of interest to all stakeholders interested in patient-oriented research in child health. The module summarizes the concept and principles of research ethics, describes examples of ethical breaches in research, explains the ethics review process in Canada, and briefly highlights ethical issues specific to patient-oriented research in child health. This module is currently under development.

The Steering Committee included two experts in knowledge translation who developed an integrated knowledge translation plan for PORCCH. The goals of the knowledge translation plan are to raise awareness of PORCCH, provide access to the curriculum, share knowledge, and build capacity in patient-oriented research in child health locally, nationally, and internationally. Target audiences include patients, families, researchers, clinicians, and other stakeholders.

## Discussion

Patient involvement in health research is an international social movement. Canada’s SPOR has already been described. In the United Kingdom, INVOLVE, which is funded by the National Institute for Health Research, has supported patient involvement in health and social care research for over 25 years [12]. Likewise, the Patient-Centered Outcomes Research Institute (PCORI) in the United States, which was established by an Act of Congress in 2010, has funded hundreds of patient-centered comparative effectiveness research projects, all driven by patient perspectives and values [13].

In the context of child health research, patient and family involvement ensures that those most affected by research findings have a voice in the research process, and that publicly funded research speaks to patient and family perspectives, needs, and outcomes [14–18]. Barriers to the engagement of patients and families in research, however, have also been identified. For researchers, barriers include unfamiliarity with participatory research as well as the additional time, funding, and resources required to meaningfully engage patients and families [19, 20]. For patients and families, unfamiliarity with the research process, language, and time-lines are considered key barriers [17, 20].

SPOR, INVOLVE, and PCORI all recommend training for researchers, patients, and families as an important tool for patient and family engagement in research [2, 12, 13]. Boote et al identified eight principles of “successful” consumer involvement in research, two of which included training and support of consumers and researchers [21]. Likewise, Frisch et al identified research knowledge and skills as core competencies for researchers and patients involved in patient-oriented research [22]. Petit-Zeman has argued that professionals and patients involved in research require training and support “*perhaps most crucially to help them understand each others’ worlds* [23].” Of note, while ‘scientific jargon’ has been identified by patients as a barrier to patient engagement, other patients have suggested that training – in the context of randomized trials – may lead to the ‘professionalization’ of patient partners [20, 24].

## Conclusions

Development of PORCCH is but one of many capacity development initiatives within the CIHR SPOR [25, 26]. Of note, patient education and training are also central to other international patient-oriented research initiatives such as PCORI, INVOLVE, and the European Patients’ Academy on Therapeutic Innovation (EUPATI) [12, 13, 27]. The PORCCH curriculum is unique; however, in that it is focused on patient-oriented research in child health and was developed to address the need for a specific curriculum, given the child- and family-centered care context and the unique ethical considerations of this population. PORCCH is a resource for patients and families to enable them to meaningfully contribute to and participate in research, thereby meeting key guiding principles of the SPOR Capacity Development framework [2]. As of April 2021, 4 months after launch, PORCCH has had over 16,800 unique visitors to the website (www.porcch.ca), with over 400 users enrolled in the modules. The website collects minimal demographic data; however, visitors to the site have come from more than twenty countries around the world. This publication is one component of the knowledge translation and dissemination plan for PORCCH. The aim is to disseminate the PORCCH curriculum, locally, nationally, and internationally.

## Data Availability

Not applicable.

## Abbreviations

CIHR: Canadian Institutes for Health Research
EUPATI: European Patients’ Academy on Therapeutic Innovation
SPOR: Strategy for Patient-Oriented Research
PORCCH: Patient-Oriented Research Curriculum in Child Health
PCORI: Patient-Centered Outcomes Research Institute
